# Characterization of the *Omija* (*Schisandra chinensis*) Extract and Its Effects on the Bovine Sperm Vitality and Oxidative Profile during *In Vitro* Storage

**DOI:** 10.1155/2020/7123780

**Published:** 2020-09-21

**Authors:** Eva Tvrdá, Jaroslav Michalko, Július Árvay, Nenad L. Vukovic, Eva Ivanišová, Michal Ďuračka, Ildikó Matušíková, Miroslava Kačániová

**Affiliations:** ^1^Department of Animal Physiology, Faculty of Biotechnology and Food Sciences, Slovak University of Agriculture in Nitra, Tr. A. Hlinku 2, 949 76 Nitra, Slovakia; ^2^BioFood Center, Faculty of Biotechnology and Food Sciences, Slovak University of Agriculture in Nitra, Tr. A. Hlinku 2, 949 76 Nitra, Slovakia; ^3^Detached Branch of the Institute of Forest Ecology-Arborétum Mlyňany, Slovak Academy of Sciences, Vieska Nad Žitavou 178, 951 52 Slepčany, Slovakia; ^4^Department of Chemistry, Faculty of Biotechnology and Food Sciences, Slovak University of Agriculture in Nitra, Tr. A. Hlinku 2, 949 76 Nitra, Slovakia; ^5^Department of Chemistry, Faculty of Science, University of Kragujevac, 34000 Kragujevac, Serbia; ^6^Department of Technology and Quality of Plant Products, Faculty of Biotechnology and Food Sciences, Slovak University of Agriculture, Tr. A. Hlinku 2, 94976 Nitra, Slovakia; ^7^Department of Ecochemistry and Radioecology, Faculty of Natural Sciences, University of SS. Cyril and Methodius in Trnava, Nám. J. Herdu 2, 917 01 Trnava, Slovakia; ^8^Department of Fruit Sciences, Viticulture and Enology, Faculty of Horticulture and Landscape Engineering, Slovak University of Agriculture, Tr. A. Hlinku 2, 94976 Nitra, Slovakia; ^9^Department of Bioenergy, Food Technology and Microbiology, Institute of Food Technology and Nutrition, University of Rzeszow, Zelwerowicza St. 4, 35601 Rzeszow, Poland

## Abstract

*Schisandra chinensis* is a woody vine native to China, Korea, and Russia, which has been used as a traditional herbal remedy to treat male infertility. As very little information is available concerning its effects on ejaculated spermatozoa, the aim of this study was to investigate the chemical, antioxidant, and antibacterial properties of the *S. chinensis* berry (*Omija*) extract followed by an assessment of its *in vitro* effects on bovine sperm function and oxidative balance. Phytochemical components of the *Omija* extract were determined by high performance liquid chromatography. The content of polyphenols, flavonoids, and carotenoids was assessed by spectrophotometric protocols. Antioxidant characteristics of the *Omija* extract were determined by the 2,2-diphenyl-1-picrylhydrazyl (DPPH) and molybdenum-reducing antioxidant power (MRAP) assays. The disc diffusion method and determination of the minimal inhibitory concentration were applied to study the antibacterial properties of *Schisandra*. Thirty semen samples were exposed to different concentrations of *Omija* (1, 5, 10, 25, 50, and 75 *µ*g/mL) for 0, 2, and 24 h. Sperm motility, mitochondrial activity, and superoxide and reactive oxygen species production, as well as total antioxidant capacity and oxidative damage to proteins and lipids were determined. Our data reveal that the *Omija* extract, particularly at a concentration range within 5–50 *µ*g/mL, exhibited dose-dependent motion-promoting and metabolism-enhancing properties, accompanied by significant antioxidant effects. We may conclude that the biomolecules present in the *Omija* extract such as schisandrins and phenolic molecules offer protection to critical sperm structures against oxidative insults and/or possible bacterial contamination, leading to a higher preservation of mammalian sperm viability and functional activity.

## 1. Introduction

Currently, artificial reproductive technologies associated with the cattle breeding industry rely almost exclusively on extended semen [1]. Unfortunately, the full potential of *ex vivo* semen handling has still not been fully exploited, as a substantial portion of male gametes lose their structural integrity or functional activity due to increased stress conditions associated with the *in vitro* environment. Spermatozoa membranes contain high amounts of polyunsaturated fatty acids (PUFAs), which serve as a preferred substrate for reactive oxygen species (ROS), leading to lipid peroxidation (LPO) [2, 3]. At the same time, the lack of substantial antioxidant protection renders spermatozoa to be highly prone to oxidative damage during semen storage. In order to palliate the effects ROS overproduction on the sperm function, several studies have tested the effects of different additives to bovine semen extenders obtaining variable results [4–6]. Generally, such additives may considerably increase the costs of insemination doses, whereas the use of natural products such as plant extracts could also provide protection to the sperm function and fertilization ability in a more cost-effective way.

*Schisandra chinensis* Baill. (*Schisandraceae*) is a woody monoecius liana native to forests of Korea, Japan, China, and Eastern Russia, which has been widely used as an essential herb in traditional oriental medicine [7]. *Schisandra* berries have all 5 of the basic flavours of salty, sweet, sour, pungent, and bitter. As such, in Korea, *Schisandra* fruits are called *Omija*, literally five-flavour berries [8].

Over the last decade, the chemistry of this plant has been extensively studied. Substantial evidence shows that *S. chinensis* and its major bioactive constituents such as polyphenols, flavonols, and lignans possess antibacterial, antioxidant, cytotoxic, and chemopreventive activities [9, 10]. Such findings may be beneficial for further understanding the pharmacological basis of *S. chinensis* as a remedy for the management or treatment of diseases accompanied by oxidative stress and metabolic disruptions, such as radiation injury, inflammation, or ischemia-reperfusion [7].

In Asia, *S. chinensis* may be found among traditional herbal remedies known to be effective in improving the sperm count, motility, and morphology [11, 12]. According to clinical studies, *Omija* exhibits antioxidant properties, and in combination with other herbs, it has been shown to improve both sperm production and activity [10–12]. Furthermore, *Omija* is famous for its ability to preserve fluids in the body, which is why it is commonly used to treat spermatorrhea, erectile dysfunction, and a decreased libido [13, 14].

Although, *in vivo*, *S. chinensis* has been frequently used to counteract male fertility issues, very little is known with respect to its *in vitro* effects on male gametes. As such, the aim of this paper was to analyse the chemical composition, antioxidant properties, and antibacterial activity of the *Omija* extract, as well as its *in vitro* effects on the functional parameters and oxidative balance of bovine spermatozoa. As a natural and cheap source of antioxidants, this extract might be a good alternative to the common additives used in bovine semen extenders.

## 2. Materials and Methods

### 2.1. Plant Material Collection and Processing

*Schisandra berries* were harvested at the Detached Branch of the Institute of Forest Ecology- Arborétum Mlyňany, Slovakia, at the end of July 2018.

For the assessment of the total phenolic and flavonoid content, as well as for the antioxidant activity evaluation, 0.2 g of the berries were freeze-dried, milled, and extracted with 20 mL 80% ethanol (Centralchem, Bratislava, Slovakia) for 24 h. After centrifugation (4000 rpm, 20 min), the supernatant was collected and used for further experiments [15]. In case of the quantification of total carotenoids, 0.5  g of dry *Omija* fruit was homogenized in the mortar with sea sand and repeatedly extracted with 10 mL acetone (Centralchem) until the sample became colourless. The extract was filtered using Whatman filter paper (Whatman, Maidstone, UK) [16].

For the high-performance liquid chromatography (HPLC) analysis, the berries were freeze-dried and milled. Methanolic extracts were prepared by adding 25 mL 80% aqueous methanol (HPLC grade; Sigma-Aldrich, St. Louis, USA) to 1 g of the sample. The mixtures were shaken on a horizontal shaker (250 rpm) at room temperature for 8 h. The samples were, then, filtered through filter paper (84 g/m^2^; Munktell & Filtrak GmbH, Bärenstein, Germany) and kept at 5°C [17].

In case of the microbiological analysis and the *in vitro* experiment, the berries were freeze-dried and crushed, weighed, and immersed in ethanol (96%; Centralchem) for two weeks at room temperature in the dark to prevent the degradation of active biomolecules. The extracts were subjected to evaporation under reduced pressure at 40°C (Stuart RE300DB rotary evaporator; Bibby Scientific Limited, Cole-Pharmer Ltd., Stone, UK, and vacuum pump KNF N838.1.2KT.45.18; KNF, Freiburg im Breisgau, Germany) to remove any residual ethanol. Crude extracts were dissolved in DMSO (dimethyl sulfoxide; Sigma-Aldrich) and adjusted to 1000 mg/mL which served as a stock solution [6].

### 2.2. Biochemical Analysis of the Extract

The total phenolic content of the *Omija* extract was measured using the Folin–Ciocalteu method [15, 16]. One hundred mL of the extract was mixed with 0.1 mL of the Folin–Ciocalteu reagent (Sigma-Aldrich), 1 mL 20% (w/v) sodium carbonate (Centralchem), and 8.8 mL distilled water. After 30 min in darkness, the absorbance was measured in triplicates at 700 nm using the Jenway 6405 UV/Vis spectrophotometer (Cole-Pharmer Ltd.). Gallic acid (25–300  mg/L; Sigma-Aldrich) was used as the standard, and the results were expressed in mg gallic acid equivalents (GAE)/g d.w. [15, 18].

The total flavonoid content was quantified with a modified method of Willett [19]. Five hundred *µ*L of the extract was mixed with 0.1 mL 10% (w/v) ethanolic solution of aluminium chloride (Sigma-Aldrich), 0.1  mL 1  M potassium acetate (Centralchem), and 4.3  mL distilled water. After 30  min incubation in the darkness, the absorbance was measured in triplicates at 415 nm with the Jenway 6405 UV/Vis spectrophotometer (Cole-Pharmer Ltd.). Quercetin (1–400  mg/L; Sigma-Aldrich) was used as the standard. The results are expressed in mg quercetin equivalents (QE)/g d.w. [16, 19].

For the assessment of total carotenoids, petroleum ether (Sigma-Aldrich) was pipetted into a separating funnel with a Teflon stopcock. The acetone extract and distilled water were added by flowing along the walls of the funnel. The mixture was allowed to separate into two phases, and the aqueous phase was discarded. The petroleum ether phase was washed twice with distilled water to remove the residual acetone. The petroleum ether phase was collected into 50 mL volumetric flasks by passing the solution through a small funnel containing 5  g anhydrous sodium sulphate (Centralchem) to remove any residual water. Subsequently, the flasks were filled up with petroleum ether. The total carotenoid content was determined from the molar absorption coefficient of *β*-carotene and expressed as mg *β*-carotene (*β*-CE)/g d.w. The measurement was performed in triplicate [16].

### 2.3. HPLC-PDA Analysis

For the quantification of dibenzocyclooctadiene lignans, a Shimadzu Prominence HPLC system consisting of an LC-20AT pump, DGU-20A degasser, CTO-20A column oven, 20 *µ*L loop, SPD-M20A PDA detector, and CBM-20A Prominence communications module was used (Kyoto, Japan). A Waters Xbridge C18 column (250 × 4.6 mm, 5 *µ*m, USA) was employed (Zellik, Belgium). The mobile phase consisted of (A) acetonitrile and water:formic acid = 100 : 0.1 (B). The flow rate was 1 mL/min, and the injection volume (loop) was 20 mL, while the column oven was adjusted at 34°C. The monitoring wavelength was set at 220 nm. The eluting conditions were as follows: isocratic at 38% A (0–26 min), linear gradient from 38% to 44% A (26–30 min), linear gradient from 44% to 48% A (30–45 min), isocratic at 48% A (48–50 min), linear gradient from 48% to 58% A (50–55 min), isocratic at 58% A (55–63 min), linear gradient from 58% to 56% A (63–64 min), isocratic at 56% A (64–85 min), linear gradient from 56% to 80% A (85–95 min), and isocratic at 80% A (95–103 min). Standards were purchased from LGC Standards SARL (Molsheim, France; Schisandrol A and B), Carbosynth (Compton, UK; Angeloylgomisin H), and Sigma-Aldrich (Gomisin G, Schisantherin A, Schisandrins A, B, and C), while HPLC-grade solvents (acetonitrile and formic acid) were obtained from J.T. Baker (Phillipsburg, USA). Water was treated in a Milli-Q water purification system (TGI Pure Water Systems, Brea, CA, USA).

In case of the quantitative analysis of the phenolic compounds, selected standards, acetonitrile (HPLC grade), and phosphoric acid (ACS grade) were purchased from Sigma-Aldrich. Double-deionized water (ddH_2_O) was prepared (0.054 mS/cm) in a Simplicity 185 purification system (Millipore SAS, Molsheim, France). Chemical composition of the *Omija* extract was determined using the Agilent 1260 Infinity high-performance liquid chromatograph (Agilent Technologies, Waldbronn, Germany) with a quaternary solvent manager coupled with a degasser (G1311B), sample manager (G1329B), column manager (G1316A), and PDA detector (G1315C). All HPLC analyses were performed on a Purosphere reverse phase C18 column (4 mm × 250 mm, 5 mm) (Merck, KGaA, Darmstadt, Germany). The mobile phase consisted of acetonitrile (gradient) (A) and 0.1% phosphoric acid in ddH_2_O (B). The gradient elution was as follows: 0–1 min isocratic elution (20% A and 80% B), 1–5 min linear gradient elution (25% A and 75% B), 5–15 min (30% A and 70% B), and 20–25 min (40% A and 60% B). The initial flow rate was 1 mL/min, and the injection volume was 1 mL. The column oven temperature was set up to 30°C, and the samples were kept at 4°C in the sample manager. The data were collected and processed using the Agilent OpenLab ChemStation software for LC 3D Systems. Prior to injection, the standard solutions and the extract were filtered through the Q-Max syringe filter (0.22 mm, 25 mm; Frisenette ApS, Knebel, Denmark).

For quantitative analysis of the extract, a mix of stock solutions (in methanol) of lignans or phenolic compounds was prepared, with the concentration of each standard compound (lignans or phenolic compounds) being 1000 *μ*g/mL. The mix of lignans or phenolic compounds was subsequently serially diluted, giving working standard solutions with concentrations ranging from 500 *μ*g/mL to 0.244 *μ*g/mL, which were used for the construction of the calibration curves. Concentrations of standard compounds in the extract were determined from the peak areas using the equation for linear regression obtained from the calibration curves. The results are expressed as mean ± S.D. of three injections [20, 21].

### 2.4. Antioxidant Activity

The free-radical scavenging activity of the extract was measured using the 2,2-diphenyl-1-picrylhydrazyl (DPPH) assay [22]. Four hundred mL of the extract was mixed with 3.6 mL DPPH solution (0.025  g DPPH, Sigma-Aldrich; in 100  mL methanol, Centralchem). The absorbance of the reaction mixture was determined at 515 nm using the Jenway 6405 UV/Vis spectrophotometer (Cole-Pharmer Ltd.). Trolox (6-hydroxy-2,5,7,8-tetramethylchroman-2-carboxylic acid; 10–100  mg/L; Sigma-Aldrich) was used as the standard, and the results are expressed in mg Trolox equivalents (TEAC)/g d.w. The measurement was performed in triplicate [16, 22].

Molybdenum-reducing antioxidant power (MRAP) of the extract was determined by the method of Prieto et al. [23] with slight modifications. The mixture of the extract (1  mL), monopotassium phosphate (2.8  mL, 0.1 M; Centralchem), sulfuric acid (6  mL, 1 M; Centralchem), ammonium heptamolybdate (0.4  mL, 0.1 M; Centralchem), and distilled water (0.8  mL) was incubated at 90°C for 120 min and, then, rapidly cooled. The absorbance was determined at 700 nm with the Jenway 6405 UV/Vis spectrophotometer (Cole-Pharmer Ltd.). Trolox (10–1000 mg/L; Sigma-Aldrich) was used as the standard, and the results are expressed in mg Trolox equivalents (TEAC)/g d.w. The assessment was performed in triplicates [16, 23].

### 2.5. Antibacterial Activity

Altogether 10 bacterial strains were tested in the study, including 5 Gram-negative bacteria (*Klebsiella pneumoniae* CCM 2318, *Pseudomonas aeruginosa* CCM 3955, *Salmonella enterica* subsp. *enterica* CCM 3807, *Shigella sonei* CCM 1373, and *Yersinia enterocolitica* CCM 5671) and 5 Gram-positive bacteria (*Bacillus cereus* CCM 7934, *Enterococcus faecalis* CCM 4424, *Listeria monocytogenes* CCM 4699, *Staphylococcus aureus* subsp. *aureus* CCM 2461, and *Streptococcus pneumoniae* CCM 4501). All tested strains were provided by the Czech Collection of Microorganisms. The bacterial suspensions were cultured in the nutrient broth (IMUNA PHARM, Šarišské Michaľany, Slovakia) at 37°C for 24 h.

#### 2.5.1. Disc Diffusion Method

A suspension of 100 *μ*L of the tested microorganism with a density of 10^6^ CFU/mL was spread on Mueller Hinton Agar (MHA; Oxoid, Basingstoke, United Kingdom). Filter paper discs of 9 mm in diameter were impregnated with 15 *µ*L of the *Omija* extract and placed on the inoculated plates. The plates were left at 4°C for 2 h and, then, incubated aerobically at 37°C for 24 h. Gentamicin (30 *μ*g/disc) was used as a positive control for the bacteria. The diameters of the inhibition zones were measured in mm. All the tests were performed in triplicates [24].

#### 2.5.2. Minimum Inhibitory Concentration

The minimum inhibitory concentration (MIC) is defined as the lowest concentration of the sample that inhibits a visible growth of microorganisms. MICs were determined by the microbroth dilution method according to the Clinical and Laboratory Standards Institute recommendation 2009 (CLSI, 2009) in Mueller Hinton broth (Biolife, Monza, Italy). *Omija* extracts were diluted in order to obtain a final concentration ranging between 0.33–682.67 mg/mL. Subsequently each plate was inoculated with a specific microbial suspension at a final density of 0.5 McFarland, and the plates were incubated at 37°C for 24 h. After incubation, the inhibition of the microbial growth was evaluated by measuring the plate absorbance at 517 nm in the absorbance microplate reader Biotek EL808 (Biotek Instruments, Winooski, USA). The plates were measured before and after the experiment. Differences between both measurements were evaluated as growth, and values exceeding 450 nm from the mean absorbance were considered as error. Wells without the extract were used as a positive control, while pure DMSO was used as a negative control. The experiment was performed in eight replicates for a higher accuracy of the MICs followed by exposure to the *Omija* extract [24].

### 2.6. *In Vitro* Experiments

#### 2.6.1. Semen Sample Collection and Culture

Semen samples (*n* = 30) were obtained from five adult Holstein Friesian breeding bulls (Slovak Biological Services, Nitra, Slovakia). One ejaculate was collected from each bull on a regular collection schedule (once a week for six consecutive weeks) using an artificial vagina. Following collection, sperm concentration and motility were assessed by phase-contrast microscopy (200x). Only ejaculates with the required quality (minimum 70% motility and concentration of 1 × 10^9^ sperm/mL) were used for the subsequent experiments. Institutional and national guidelines for the care and use of animals were followed, and all experimental procedures were approved by the State Veterinary and Food Institute of Slovak Republic (no. 3398/11-221/3) and Ethics Committee.

Each sample was diluted in PBS (Dulbecco's Phosphate Buffer Saline without calcium chloride and magnesium chloride; Sigma-Aldrich) containing various concentrations of the *Omija* extract (1, 5, 10, 25, 50 and 75 *μ*g/mL) using a dilution ratio of 1 : 40. The samples were cultured at laboratory temperature (22°C–25°C). After culture periods of 0, 2, and 24 h, spermatozoa motility, mitochondrial activity, ROS generation, and superoxide production were assessed in each group. Furthermore, an aliquot of each group was centrifuged at 5000 rpm, 25°C for 10 min, the media were removed, and the resulting pellet was sonicated at 28 kHz for 30 s on ice using RIPA buffer (Sigma-Aldrich) with protease inhibitor cocktail for mammalian cell and tissue extracts (Sigma-Aldrich). Subsequently, the samples were centrifuged at 10,000 rpm at 4°C for 15 min to purify the lysates from the residual cell debris [5]. The resulting supernatants comprising the intracellular content were stored at −20°C for the assessment of the total antioxidant capacity, as well as oxidative damage to the proteins and lipids.

#### 2.6.2. Sperm Motility Analysis

Spermatozoa motion characteristics were assessed using the computer-aided sperm analysis (CASA; Version 14.0 TOX IVOS II.; Hamilton Thorne Biosciences, Beverly, USA). With the objective to avoid false-positive results, the samples were stained using the IDENT Stain, a DNA-specific dye based on Hoechst bisbenzimide (Hamilton Thorne Biosciences), and analysed under fluorescent illumination.

The system was set up as follows: frame rate- 60 Hz; minimum contrast- 20; static head size- 0.25–5.00; static head intensity- 0.40–2.00; static elongation- 20–100; default cell size- 4 pixels; and default cell intensity- 40. Ten *μ*L of each sample was placed into the Makler counting chamber (depth 10 *μ*m, 37°C; Sefi Medical Instruments, Haifa, Israel) and immediately assessed for sperm motility (MOT; percentage of motile spermatozoa; motility >5 m/s; %). Ten microscopic fields were subjected to each analysis to comprise, at least, 300 cells [5].

#### 2.6.3. Mitochondrial Activity

The sperm mitochondrial activity was evaluated using the colorimetric metabolic activity (MTT) test, which is based on the conversion of a yellow tetrazolium salt (3-(4,5-dimetylthiazol-2-yl)-2,5-diphenyltetrazolium bromide; MTT) to blue formazan particles by mitochondrial succinate dehydrogenase of intact mitochondria within living cells. The tetrazolium salt (Sigma-Aldrich) was dissolved in PBS at 5 mg/mL. Twenty *μ*L of the tetrazolium solution was added to each sperm suspension. After a 2 h incubation (shaker, 37°C, 95% air atmosphere, 5% CO_2_), the formazan crystals were dissolved in 80 *μ*L acidified (0.08 mol/L HCl; Centralchem) isopropanol (Centralchem). The optical density was determined at a wavelength of 570 nm against 620 nm as the reference using a Multiskan FC microplate photometer (Thermo Fisher Scientific Inc., Waltham, USA). Data are expressed as percentage of the control set to 100% [5].

#### 2.6.4. Quantification of the Superoxide Production

The nitroblue tetrazolium (NBT) test was used to quantify the intracellular formation of the superoxide radical, by assessing blue NBT formazan deposits, generated by the reduction of the membrane-permeable, yellow-coloured, nitroblue tetrazolium chloride (2,20-bis(4-Nitrophenyl)-5,50-diphenyl-3,30-(3,30-dimethoxy-4,40-diphenylene) ditetrazolium chloride; Sigma-Aldrich) by the superoxide radical. The NBT salt was dissolved in PBS containing 1.5% DMSO (Sigma-Aldrich) to a final concentration of 1 mg/mL and added to the cells (100 *μ*L per well). After a 1 h incubation (shaker, 37°C, 95% air atmosphere, 5% CO_2_), the cells were washed twice with PBS and centrifuged at 1250 rpm for 10 min. Lastly, the cells and formazan crystals were dissolved in 2 mol/L KOH (potassium hydroxide; Centralchem) in DMSO. The optical density was determined at a wavelength of 620 nm against 570 nm as the reference by using a Multiskan FC microplate photometer (Thermo Fisher Scientific Inc.). Data are expressed in percentage of the control set to 100% [6].

#### 2.6.5. ROS Generation

ROS production was assessed by the chemiluminescence assay using luminol (5-amino-2, 3- dihydro-1, 4-phthalazinedione; Sigma-Aldrich) as the probe [25]. The tested specimens consisted of luminol (10 mL, 5 mmol/L) and 400 mL of the control or experimental sample. Negative controls were prepared by replacing the sperm suspension with 400 *μ*L of each culture medium. Positive controls included 400 *μ*L of each medium, 10 *μ*L luminol, and 50 *μ*L hydrogen peroxide (H_2_O_2_, 30%; 8.8 M; Sigma-Aldrich). Chemiluminescence was measured on 48-well plates in fifteen 1 min cycles using the Glomax Multi^+^-combined spectrofluoroluminometer (Promega Corporation, Madison, USA) [5]. The results are expressed as relative light units (RLU)/s/10^6^ sperm.

#### 2.6.6. Total Antioxidant Capacity (TAC)

An improved enhanced chemiluminescence antioxidant assay using horseradish peroxidase conjugate and luminol was used to study the total antioxidant capacity of the sample [26]. A concentration range of 5–100 mmol/L Trolox (Sigma-Aldrich) was used as the standard, while a signal reagent consisting of 0.1 mol/L Tris-HCl (Sigma-Aldrich), 12 mol/L H_2_O_2_ (Sigma-Aldrich), 41.8 mmol/L 4-iodophenol (Sigma-Aldrich), and 282.2 mmol/L luminol (Sigma-Aldrich) was used to induce the chemiluminiscent reaction. Chemiluminescence was measured on 96-well plates in 10 cycles of 1 min using the Glomax Multi^+^-combined spectrofluoroluminometer (Promega Corporation). The results are expressed as *μ*mol Trolox Eq./mg protein.

#### 2.6.7. Assessment of Oxidative Damage

Carbonyl group quantification was performed using the traditional 2,4-dinitrophenylhydrazine (DNPH) method. Briefly, 1 mL of the pretreated sample solution was added to 1 mL DNPH (10 mM in 2N HCl; Sigma-Aldrich), mixed, and incubated for 1 h in the dark at 37°C. After the addition of 1 mL of trichloroacetic acid (20% w/v; Sigma-Aldrich), the mixture was incubated at 4°C for 10 min before centrifugation at 5000 rpm for 15 min. The supernatant was discarded without disturbing the pellet that was washed three times with 1 mL ethanol/ethyl acetate (1/1; v/v; Sigma-Aldrich) to remove the free DNPH reagent. The sample pellet was resuspended in 1 mL of 6 M guanidine-HCl (Sigma-Aldrich) before absorbance measurement at 360 nm using the Cary 60 spectrophotometer (Agilent Technologies). The molar absorption coefficient of 22,000 1/M.cm was used to quantify the concentration of protein carbonyls groups. Protein carbonyls are expressed as nmol/mg protein [27].

LPO expressed through malondialdehyde (MDA) production was assessed with the help of the TBARS assay, modified for a 96-well plate. Each sample was treated with 5% sodium dodecyl sulphate (SDS; Sigma-Aldrich) and subjected to 0.53% thiobarbituric acid (TBA; Sigma-Aldrich) dissolved in 20% acetic acid adjusted with NaOH (Centralchem) to pH 3.5 and subsequently boiled at 90°C–100°C for 1 h. Following boiling, the samples were placed on ice for 10 min and centrifuged at 5000 rpm for 10 min. The supernatant was used to measure the end-product resulting from the reaction of MDA and TBA under high temperature and acidic conditions at 540 nm with the help of the Multiskan FC microplate photometer (Thermo Fisher Scientific Inc.) [5]. The MDA concentration is expressed as mmol/g protein.

Protein concentration was quantified using the DiaSys Total Protein (DiaSys, Holzheim, Germany) commercial kit and the semiautomated clinical chemistry photometric analyser RX Monza (Randox, Crumlin, UK). The measurement is based on the Biuret method, according to which copper sulphate reacts with proteins to form a violet blue colour complex in an alkaline solution, and the intensity of the colour is directly proportional to the protein concentration when measured at 540 nm [5].

### 2.7. Statistical Analysis

Statistical analysis was carried out using the GraphPad Prism program (version 6.02 for Windows; GraphPad Software, La Jolla, CA, USA, http://www.graphpad.com). Descriptive statistical characteristics (mean, standard deviation) were evaluated at first. One-way ANOVA was used for specific statistical evaluations. Dunnett's test was applied as a follow-up test to ANOVA, based on a comparison of every mean to a control mean and computing a confidence interval for the difference between the two means. The level of significance was set at ^∗∗∗^-*p* < 0.001, ^∗∗^-*p* < 0.01, ^*∗*^-*p* < 0.05.

## 3. Results and Discussion

Bovine semen samples used for artificial insemination are usually diluted with commercial extenders, with a concomitant reduction of seminal plasma. Nevertheless, the importance of seminal plasma for the sperm survival has been emphasized in numerous studies, mostly due to the presence of a variety of molecules with ROS-scavenging and antioxidant properties [28]. As such, the addition of antioxidants to semen extenders has become a standard step in artificial reproduction technologies, although it often increases the final cost of semen doses [4, 6, 29, 30]. As an alternative, medicinal plants and their products are increasingly recognized worldwide as an alternative source of efficient and cost-effective biomolecules that could be used as supplements during sperm storage [5, 31–33].

Although *S. chinensis* has been used for decades to treat male reproductive dysfunction, little is known about its impact on ejaculated spermatozoa. As such, this study attempted to shed more light on the chemicobiological properties of the *Omija* extract followed by the assessment of its *in vitro* impact on the functional activity and oxidative profile of bovine spermatozoa.

The benefits of *Schisandra* have recently gained scientific attention as its products are a notable source of bioactive molecules, exhibiting a wide range of protective or beneficial effects on human and animal health [7, 9]. A compelling body of evidence reveals that some of these effects may be related to an intricate chemical structure and antioxidant activity of such compounds. Polyphenols and flavonoids dispose a remarkable ROS-scavenging ability and are reported to prevent chronic or degenerative diseases [34].

### 3.1. Biochemical Analysis

Data collected from the biochemical assessment of the *Omija* extract are presented in Table 1. According to the Folin–Ciocalteu method, the total polyphenolic content of the extract was 16.52 ± 1.34 mg GAE/g d.w. The extract contained 2.66 ± 0.31 mg QE/g d.w. of flavonoids and 0.15 ± 0.02 mg *β*-CE/g d.w.

The biochemical assessment of the *Omija* extract revealed that the berry is a rich source of phytochemicals, particularly phenolic compounds, complementing the findings by Ivanišová et al. [35]. Mocan et al. [36] elected a similar experimental approach to assess the general composition of *S. chinensis* leaves and fruits. Their analysis showed that the fruits contained lower amounts of polyphenols (9.20 ± 0.43 mg/g d.w.) when compared to our data. Inversely, according to Wang et al. [37], the amount of polyphenols was more abundant in their samples (26.79 ± 17.06 mg/g d.w.) in comparison to our specimens.

On the other hand, the total flavonoid content detected in our samples was lower in comparison with other studies [36, 37], although we used quercetin as a standard for the quantification of the flavonoids, whereas resveratrol or catechin were used by Mocan [36] or Wang [37], respectively. The discrepancies in the overall concentrations of polyphenols may be explained by different extraction solvents and/or protocols selected for each study. An important point to be considered with this regard is that the presence and amount of biomolecules is highly dependent on a number of environmental factors, such as the geographical location, climate, soil type, harvesting procedures, and plant material processing [37].

The presence of carotenoids in *S. chinensis* has not been studied in detail before. Our results suggest that carotenoids comprise a relatively small fraction of the *Omija* extract, agreeing with Ivanišová et al. [35] who state that the concentration of carotenoids in *S. chinensis* was lower in comparison to sea buckthorn or goji. As such, we may hypothesize that the biological activity of *S. chinensis* may not be attributed to tetraterpenoids. Nevertheless, it would be of interest to examine the specific carotenoids and their contribution to the complex characteristics of the *Omija* berry.

### 3.2. High-Performance Liquid Chromatography (HPLC)

A straightforward and accurate HPLC-photodiode array (HPLC-PDA) detection method was used for the identification and quantification of dibenzocyclooctadiene lignans and phenolic molecules present in the *Omija* extract. The quantitative determination was performed by the external standard method, and the concentrations of the identified compounds are provided in Table 2.

The main lignan detected in the extract was schisandrol A (9280.00 ± 11.00 *µ*g/g d.w.). From the analysed phenolic acids, protocatechuic acid (259.50 ± 9.58 *µ*g/g d.w.), gallic acid (56.80 ± 2.81 *µ*g/g d.w.), and chlorogenic acid (44.90 ± 1.98 *µ*g/g d.w.) were the most abundant. Three flavonoid glycosides (rutin, quercitrin, and isoquercitrin) and two flavonoid aglycones (myricetin and quercetin) were found in the extract. Among the flavonoid glycosides, rutin was the main molecule (131.25 ± 5.85 *µ*g/g d.w.) and myricetin was the dominant compound among free aglycones (35.62 ± 0.69 *µ*g/g d.w.). Furthermore, (+) catechin was identified as the only flavanol present in the *Omija* extract (Table 2).

A number of studies have focused on the chemical characterization of *S. chinensis* and its products. Similarl to our data, the phytochemical investigation led by Mocan et al. [36] revealed that the most notable phenolic acid present in *Schisandra* leaves and fruits was chlorogenic acid. A more detailed analysis by HPLC-mass spectrometry [38] also confirmed the presence of protocatechuic and neochlorogenic acids. Correspondingly, the study revealed that gentisic and p-coumaric acids were present in very low quantities. Furthermore, Szopa and Ekiert [39] also detected protocatechuic, p-hydroxybenzoic, salicylic, and syringic acid in *Schisandra* berries collected from *in vitro* cultures. In a subsequent study by Szopa et al. [40], chlorogenic, gallic, and vanilic acid were identified in the fruits collected from *Schisandra in vitro* agar systems using an HPLC-DAD approach. Interestingly, none of the studies claims to have detected *α*-resorcylic or ferulic acid, although similarl to our report, caffeic acid was not detected in the *Omija* berries. On the other hand, (E)-cinnamic acid was identified by Sladkovsky et al. [41].

Among the identified polyphenols, Mocan et al. [36] reported that rutin, quercetin, and isoquercitrin were among the predominant representatives in *Schisandra*, although all three biomolecules were present in lower amounts in comparison to our plant material. On the contrary, hyperoside was not detected in our extract. Inversely, we did detect quercitrin in a relatively high amount, as did Szopa et al. [40]. Furthermore, notable quantities of (+) catechin, myricetin, and syringin were found in our *Omija* specimens. Interestingly, Sladkovsky et al. [41] used a reversed-phase HPLC separation method with UV spectrophotometric detection for the assessment of the vegetative organs of *S. chinensis*, enabling the identification of kaempferol, which was not identified in our case. These discrepancies may be explained by a varying standards composition selected for the chemical analysis, as well as by a different primary extraction method, time, and the solvent used. Other factors, both geographical- and harvesting-related, must be taken into consideration as well.

Lignans are considered to be primarily responsible for the beneficial properties of the *Omija* berry, although these may be found also in the shoots, leaves, and *in vitro* cultures of *Schisandra*. The first ever study to point out the presence of bioactive lignan molecules in *Schisandra* was published by Avula et al. [42] in 2005. Their HPLC approach determined nine lignans in *S. chinensis* samples collected from different regions in South Korea. A similar report published by Hu et al. [43] identified eleven lignans in 22 *S. chinensis* samples collected from different geographical locations in China. As in our study, schisandrins, schizandrols, and gomisins were the predominant lignans detected in the *Omija* berries in both cases. Furthermore, similar amounts of schisandrols A and B, as well as schisandrin A as the predominant bioactive lignans determined in *Schisandra* fruits collected from Inje and Mungyeong, Korea, were reported by Sowndhararajan et al. [44]. Our data show that, among the dibenzocyclooctadiene lignans present in the *Omija* berry, schisandrol A was the dominant compound while the content of schisandrol B, deoxychisandrin, and gomisins was significantly lower, agreeing with reports evaluating *S. chinensis* fruit specimens collected in China [45], Korea [46], Poland [47], and Romania [38].

### 3.3. Antioxidant Activity

The DPPH assay revealed that the free-radical scavenging activity of the extract was 5.93 mg TEAC/g d.w. Moreover, according to the MRAP assay, the antioxidant activity of the *Omija* extract was 140.52 mg TEAC/g d.w (Table 3).

Over the past decade, *S. chinensis* and its products have become a spotlight of scientific attention, particularly because of a prominent antioxidant behavior, which is strongly related with their chemical composition. *Schisandra* is known to contain abundant amounts of polyphenols and flavonoids, which are reflected in substantial ROS-scavenging properties [44, 46]. The results obtained from the DPPH and MRAP assays match the report of Ivanišová et al. (5.85 ± 0.01 mg TEAC/g d.w. and 148.87 ± 18.21 mg TEAC/g d.w., respectively) stating that the antioxidant activity of the *Omija* berry was comparable to mulberry (in the case of DPPH) or acai (with respect to MRAP). Liu et al. [48] reported that essential oils collected from the *Omija* berry exhibited a higher DPPH reactivity in comparison with essential oils prepared from the *S. chinensis* seeds. Furthermore, Wang et al. [37] revealed that the ethanol extract and ethyl acetate fraction of *Omija* berries exhibited a high potential in scavenging DPPH radicals, although it was revealed that *S. sphenanthera* performed better in comparison to *S. chinensis*. A slightly higher DPPH antioxidant capacity was observed by Mocan et al. [36] (7.80 ± 0.55 mg QE/g d.w.) which correlated well with the Trolox equivalent antioxidant capacity (TEAC) assay based on a similar experimental principle (ROS scavenging by electron transfer mechanism). In our case, the differences in the antioxidant activity of the *Omija* extract may be explained by the fact that the DPPH and MRAP assays follow a different experimental foundation. While in the DPPH assay, ROS-quenching molecules are predominantly represented by mineral compounds, vitamins, and poylphenols, in the MRAP protocol, the reduction of Mo^VI^ to Mo^V^ is primarily executed by polyphenols and carotenoids. As such, it is recommendable to assess the antioxidant activity of a sample by using more than one method [35].

A more physiological approach to assess the antioxidant activity of ethanolic extracts prepared from *Schisandra* was elected by Mocan et al. [36] by monitoring the effects of the extracts on the peroxidase activity of cytochrome c and the formation of lipid-conjugated dienes. The antioxidant capacity of the tested extracts, reflected in the delay of LPO, confirmed the ability of the biomolecules of *S. chinensis* to prevent and/or delay oxidative damage, which was, furthermore, validated by a good correlation with DPPH and TEAC results. Moreover, Singha and Das [49] reported that the water extract of the *Omija* skin and pulp exhibited a potent peroxyl radical-scavenging capacity.

### 3.4. Antibacterial Activity

The antibacterial activity of the *Omija* extract varied depending on the microorganism (Table 4). The disc diffusion method showed different antimicrobial effects of *S. chinensis* against two different groups of bacteria. The best antibacterial activity was found against Gram-positive bacteria. The most sensitive bacterial strain was *S. pneumoniae*.

The MIC 50 values obtained from the antimicrobial tests ranged from 64.2 to 170.7 *µ*g/mL (Table 5), while the MIC 90 ranged from 72.2 to 192.9 *µ*g/mL. The results showed that *E. faecalis* was more sensitive to the *Omija* extract with an MIC 50 value of 64.2 *µ*g/mL and MIC 90 of 85.3 *µ*g/mL, respectively, followed by *L. monocytogenes* with an MIC 50 value of 64.2 *µ*g/mL and MIC 90 of 72.2 *µ*g/mL.

Semen quality is continuously endangered by numerous factors, among which bacterial contamination is a prominent one. The ever-present bacteria have practically unlimited possibilities to contaminate semen during collection, processing, and storage. It is known that bacteriospermia contributes significantly to the loss of sperm motility, morphological alterations to spermatozoa, acrosome dysfunction and oxidative damage to biomolecules critical to the structural integrity, and functional activity of male gametes [50]. The use of antibiotics is a popular option in semen processing procedures; nevertheless, it is necessary to take into account the negatives of such practice, particularly the ever-increasing resistance of common bacterial species and possible toxic effects of the antibiotic on the functional manifestations of the male reproductive cell. A modern trend in the development of new semen extenders is the application of alternative supplements that could contribute to the reduction of sperm damage resulting from *ex vivo* semen processing. A systematic screening of natural resources also represents a continual effort by the scientific community to discover new substances with the potential to replace antibiotics in animal production [51, 52]. Based on a convincing evidence that the biomolecules present in *Schisandra* possess antibacterial properties, we studied the efficiency of the *Omija* extract to inhibit the growth of common pathogens (*K. pneumoniae*, *S. sonei*, and *L. monocytogenes*), as well as bacteria previously detected in semen samples collected from rabbits, bulls, and boars (*B. cereus*, *E. faecalis*, and *P. aeruginosa*) [52–54].

The *Omija* extract showed an efficient antibacterial activity against all targeted bacteria. The growth of all bacterial strains was inhibited by the extract, with a maximal inhibition zone of 11 mm in case of *S. pneumoniae*, followed by *E. faecalis* and *B. cereus*. The *Omija* extract was particularly effective against Gram-positive bacteria, agreeing with Mocan et al. [36] and Chen [55], although it must be noted that *E. coli* and *S. typhimurium* were nonsensitive to the *S. chinensis* fruits extract as observed by Mocan et al. [36]. As *E. coli* and *S. typhimurium* are Gram-negative, we may speculate that Gram-negative bacteria may be more resistant because of their impenetrable cell wall. The assessment of the MIC values revealed that *E. faecalis*, *L. monocytogenes*, *S. aureus*, and *B. cereus* were the most sensitive strains towards the *Omija* extract. Again, the collected data suggesting that the extract is more effective against Gram-positive bacteria complement the previous findings published by Hussain et al. [56], Teng and Lee [57], and Mocan [36], hypothesizing that Gram-positive bacteria are more sensitive to plant essential oils than Gram-negative bacteria. It has been previously reported that bioactive compounds present in plant products such as resveratrol or quercetin have the ability to provide protection to animal spermatozoa against deleterious changes to their structure and function as a result of bacterial contamination [52]. Therefore, *S. chinensis* could become an interesting source of alternative supplements to semen extenders providing higher protection of male gametes against bacteriospermia.

### 3.5. Assessment of the Sperm Vitality and Oxidative Profile

A summary of results on the motility (MOT) assessment is shown in Table 6. The CASA analysis revealed a continuous decrease in the sperm motion during the *in vitro* culture. No significant differences were found in the MOT behavior among the control and experimental groups at 0 h and 2 h. Nevertheless, at the end of the experiment (time 24 h), the CASA assessment revealed a significantly slower decrease of sperm motion in the groups subjected to 1–50 *µ*g/mL extract when compared to the control (*p* < 0.001).

**Figure 1:**
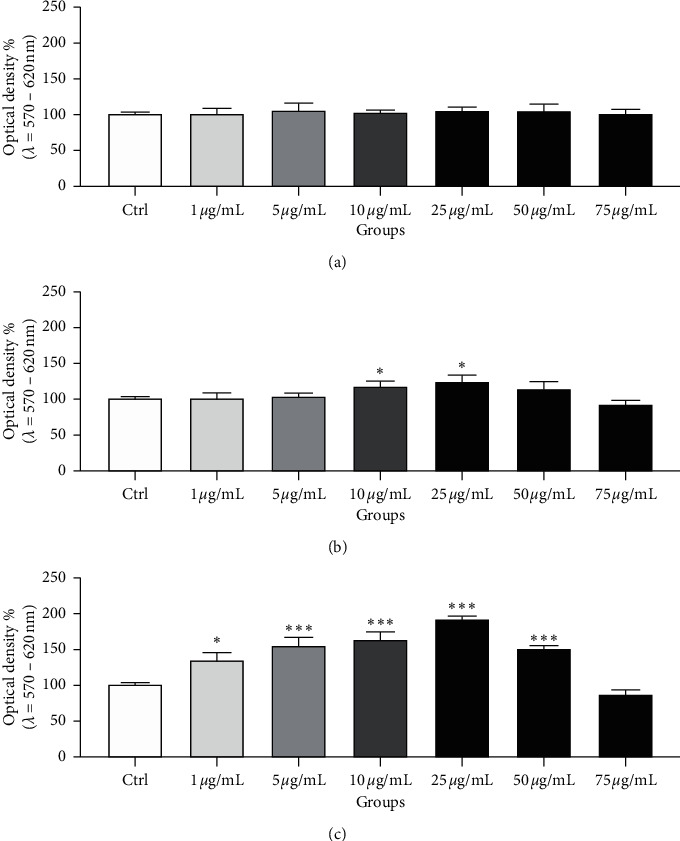
Spermatozoa mitochondrial activity (%) in the absence (Ctrl) or presence of the *Omija* extract in different time periods. Each bar represents mean (±S.D.) optical density as the percentage of the control, which symbolizes 100%. The data were obtained from six independent experiments. ^*∗∗∗*^-*p* < 0.001; ^*∗*^-*p* < 0.05. (a) Mitochondrial activity 0 h. (b) Mitochondrial activity 2 h. (c) Mitochondrial activity 24 h.

The mitochondrial metabolic (MTT) test revealed early at time 2 h that the presence of 10 and 25 *µ*g/mL *Omija* extract caused a significant increase of the sperm mitochondrial activity (*p* < 0.05; Figure 1). The assessment at 24 h revealed a significant mitochondrial promoting effect of *Omija* supplementation to the sperm culture, particularly at concentrations ranging between 1 and 50 *µ*g/mL (*p* < 0.05 with respect to 1 *µ*g/mL; *p* < 0.001 in case of 5–50 *µ*g/mL) when compared to the untreated control. On the other hand, the presence of 75 *µ*g/mL *Omija* extract exhibited inhibitory effects on the viability of bovine spermatozoa, particularly at later culture times, as revealed by a significantly lower mitochondrial activity in comparison to the untreated control at time 24 h (*p* < 0.05).

**Figure 2:**
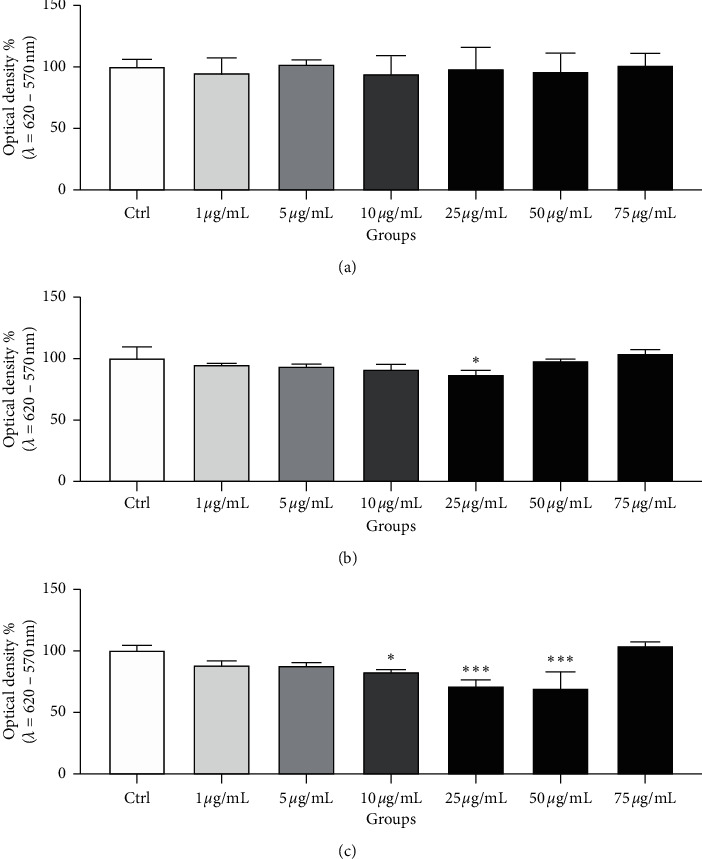
Intracellular superoxide production (%) by bovine spermatozoa in the absence (Ctrl) or presence of the *Omija* extract in different time periods. Each bar represents mean (±S.D.) optical density as the percentage of the control, which symbolizes 100%. The data were obtained from six independent experiments. ^*∗∗∗*^-*p* < 0.001; ^*∗*^-*p* < 0.05. (a) Superoxide production 0 h. (b) Superoxide production 2 h. (c). Superoxide production 24 h.

The nitroblue tetrazolium (NBT) test confirmed that the extract obtained from the *Omija* berries possesses superoxide-quenching properties (Figure 2). A significant enhancement of the superoxide balance became evident after 2 h of the *in vitro* culture with respect to 25 *μ*mol/mL extract (*p* < 0.05), while at time 24 h, the concentration range of the extract with significant protective effects on the intracellular superoxide milieu increased to 10–50 *μ*g/mL when compared to the control (*p* < 0.05 in case of 10 *μ*g/mL; *p* < 0.001 with respect to 25 and 50 *μ*g/mL).

A similar trend was observed with respect to the assessment of the impact of the *Omija* extract on the overall ROS production ROS using luminometry and luminol as the probe. A significantly lower ROS production was recorded after 2 h of exposition to 5–50 *μ*g/mL extract when compared to the control group (*p* < 0.05 in case of 5, 10, and 50 *μ*g/mL; *p* < 0.001 with respect to 25 *μ*g/mL). Long-term antioxidant effects of *S. chinensis* were confirmed by the 24 h assessment, as the ROS generation was significantly lower in the experimental groups subjected to a concentration range 1–50 *μ*g/mL extract in comparison to the control (*p* < 0.01 in relation to 1 and 50 *μ*g/mL; *p* < 0.001 with respect to 5, 10, and 25 *μ*g/mL; Table 7).

The assessment of the total antioxidant status revealed a significant increase of this parameter in the experimental samples subjected to 1–75 *μ*g/mL extract (*p* < 0.01), probably due to the enrichment of the intracellular milleu by the ROS-quenching molecules present in *Omija*. This rapid increase was followed by a slow decrease of the total antioxidant capacity; however, the parameter was still significantly higher in the samples exposed to 1–75 *μ*g/mL extract after 24 h (*p* < 0.01; Table 8).

To evaluate the ability of *S. chinensis* to provide protection against oxidative insults to proteins and lipids, we focused to quantify the amount of protein carbonyls, as well as malondialdehyde, as primary end-products of protein and lipid oxidation following exposure of male reproductive cells to the *Omija* extract. Both examinations revealed that all chosen concentrations of *Omija* exhibited protective effects on the protein and lipid molecules early on into the *in vitro* culture (time 2 h; Table 9 and 10), and maintained these beneficial effects throughout the later assessment time resulting into a significantly lower occurence (*p* < 0.001) of both protein carbonyls (Table 9) and malondialdehyde (Table 10) in all experimental groups when compared to the control.

In this study, the *Omija* extract supplemented to bovine spermatozoa acted as an effective motion-promoting and metabolism-enhancing molecule, significantly improving the motility and mitochondrial activity of the male gametes.

Mitochondrial metabolism is a key factor supporting essential sperm functions; hence, their integrity and functionality is critical to ensure the fertilization success [58]. At the same time, mitochondria are the primary source of ROS, small amounts of which are necessary for the male gamete to acquire the fertilizing capability. On the other hand, mitochondrial malfunction followed by ROS overgeneration may lead to detrimental alterations in the male reproductive cell [59]. ROS can, therefore, exhibit both physiological and pathological effects on sperm vitality, which is why an adequate oxidative balance is essential to maintain a satisfactory reproductive performance in males [58].

The mitochondrial complexes I and III have been identified as primary ROS producers [59]. When superoxide is generated, superoxide dismutase catalyzes its dismutation to hydrogen peroxide, which may be converted to highly reactive hydroxyl radicals via the Fenton reaction. Subsequently, ROS may oxidize thiol groups (-SH) of the mitochondrial permeability transition pores, leading to their opening and an increased membrane permeability through LPO [59, 60].

Chen et al. [61] showed that schisandrins, major components of the *Omija* berries inhibited the depolarization of mitochondrial membranes and preserved the mitochondrial structural integrity by increasing the resistance to Ca^2^-induced permeability transition and by decreasing the release of cytochrome c, hence preventing mitochondrial swelling. Furthermore, it was found that schisandrins balanced the energy metabolism and enhanced the mitochondrial antioxidant status, as assessed by the levels of reduced glutathione, alpha-tocopherol, and superoxide dismutase [62]. At the same time, catechin and quercetin found in our extract have the ability to modulate the activity of complex I of the electron transport chain, leading to an increased ATP production and a reduced oxidative damage [20]. Last but not the least, Parihar et al. [63] and Semaming et al. [64] reported that gallic and protocatechuic acid can act as electron acceptors at the mitochondrial complex I, resulting in an increased ATP production and regulation of energy metabolism in mammalian cells. Summarizing all the abovementioned observations, it may be feasible to say that the major phytochemicals present in the *Omija* extract offer protection to the structural stability and function of sperm mitochondria and prevent their disruption caused by oxidative outbursts, leading to a more effective preservation of the sperm motility.

To our knowledge, two studies exist on the effects of biologically active molecules isolated from *Schisandra* on male reproduction. In an *in vivo* study, Zhang et al. [21] explored the efficiency of *S. chinensis* polysaccharide (SCP) on cyclophosphamide-induced dyszoospermia of rats and its effects on reproductive hormones. Following an intragastric injection of SCP, the sperm concentration and motility increased and teratozoospermia rates decreased, while testosterone concentrations in the testicular homogenate increased. Histological analysis revealed that the pathological injury in the testicular tissue was improved in all SCP groups. SCP showed obvious therapeutical effects on dyszoospermia in rats, and its mechanisms might be associated with recovering the regulation function of the hypothalamic-pituitary-gonadal axis. Zhao et al. [65] chose to study the *in vitro* effects of SCP on mice spermatozoa subjected to heat-induced stress. Following exposure to 0.2, 0.4, and 0.8 mg/mL SCP for 30 min and 42°C, it was revealed that spermatozoa motility was significantly increased particularly in the case of 0.4 and 0.8 mg/mL SCP in comparison to the positive group. The authors suggested that the protective effects of SCP may be linked to the antioxidant properties of the *Omija* biocomponents.

Our nitroblue tetrazolium and luminometric data highlight the potent ROS-scavenging abilities of biomolecules found in the *Omija* berry, supporting Mocan et al. [36] who hypothesize that the phenolic structure of biologically active components of *Omija* contributes to their antioxidant action by removing the superoxide, singlet oxygen, peroxide, hydrogen peroxide, and the hydroxyl radical. Giovannini et al. [66] reported that polyphenols upregulated the glutathione cycle, which is crucial for a normal cell defense during oxidative stress. Moreover, according to Chae et al. [67], flavonoids such as rutin, quercetin, and myricetin have the ability to restore and modulate the activity of major antioxidant enzymes during increased oxidative pressure. Thanks to such properties, we may suggest that the *Omija* extract could contribute to a higher stability of the oxidative milieu of male reproductive cells exposed to a stressful *in vitro* environment.

It has been previously reported that *in vitro* sperm processing may be associated with alterations to the mobility of -SH-containing proteins in the sperm membrane. As -SH groups are under redox control, changes in the redox properties of the membrane can be linked to ROS overproduction that occurs during sperm preservation [68]. At the same time, ROS are able to interact with or modify diverse protein structures and functions, leading to protein oxidation, aggregation, fragmentation, or lysis [69]. Protein carbonyls are a commonly observed product of oxidative damage to peptides crucial for the survival of male reproductive cells. Our data reveal that the *Omija* extract was able to diminish the amount of protein carbonyls in bovine spermatozoa probably by a direct ROS-quenching ability, hence preventing the residual ROS from interacting with the structure and/or function of the protein molecules present in the spermatozoon.

Membranous structures of the sperm cell are exceptionally sensitive to ROS overproduction, leading to alterations in the kinetics of the intramembranous enzymes and decreased membrane fluidity [2]. Our experimental data suggest that the *Omija* extract has the ability to maintain the sperm membrane intact when compared to the untreated control. Since malondialdehyde (MDA) is produced as an end product of LPO, its amounts are a suitable indicator of the damage caused to lipid membranes. Correspondingly, it was shown that the vast majority of phytochemicals present in *Omija* have the ability to protect the structure and function of vital cellular components against oxidative damage through their antioxidant properties, including directly scavenging excessive ROS or limiting their availability, thus protecting the lipid components of cellular and mitochondrial membranes [62, 65, 67]. As such, our data suggest that *Omija* exhibits the ability to, at least partially, prevent oxidative insults to critical biomolecules of male reproductive cells and complement earlier studies reporting on its antioxidant roles in mammalian cells by means of measuring diverse oxidative biomarkers such as the activity of superoxide dismutase and the extent of LPO [65, 70, 71].

The most significant limitation of this study is the complex nature of the *Omija* extract. Plant extracts are intricate substances containing a number of components which may exhibit significant ROS-quenching and protective properties in synergy. Knowledge on the individual impact of the different phytochemicals present in the *Omija* extract on sperm characteristics is sparse at present and needs to be evaluated further. As such, the next step in this investigation shall be to separate the components previously characterized by HPLC individually, in order to assess their specific contribution to the protective and antioxidant effects of the *Omija* extract. Moreover, the full potential of the *Omija* extract may be confirmed by testing its effects on the sperm function and fertilization ability under induced oxidative stress conditions.

## 4. Conclusions

In conclusion, our results emphasize on the protective and antioxidant properties of the *Omija* extract against oxidative damage to bovine spermatozoa. This behavior could be attributed to polyphenols, lignans, and flavonoids which represent the main biologically active compounds of the extract. Further research is necessary to understand specific characteristics of individual *S. chinensis* components underlying the beneficial effects of the *Omija* extract on mammalian gametes, as well as the potential therapeutic use and safety of this extract for the preservation of bovine semen [71].

## Figures and Tables

**Table 1:** Biochemical characterization of the *Omija* extract.

Parameter	Concentration

Total phenolic content	16.52 ± 1.34 mg GAE/g d.w.
Total flavonoid content	2.66 ± 0.31 mg QE/g d.w.
Total carotenoid content	0.15 ± 0.02 mg *β*-CE/g d.w.

The measurements were performed in triplicate. Mean ± S.D.

**Table 2:** Chemical composition of the *Omija* extract.

Dibenzocyclooctadiene lignans	Retention time (min)	Concentration (*µ*g/g d.w.)
Schisandrin A	19.92	580.00 ± 70.00
Schisandrin B	29.35	140.00 ± 10.00
Schisandrin C	39.95	110.00 ± 10.00
Schisandrol A	45.54	9280.00 ± 110.00
Schisandrol B	51.99	1680 ± 30.00
Angeloylgomisin H	65.95	1890.00 ± 40.00
Gomisin G	76.35	3120.00 ± 90.00
Schisantherin A	86.55	260.00 ± 20.00
Phenolic compounds	Retention time (min)	Concentration (*µ*g/g d.w.)
Gallic acid	2.87	56.80 ± 2.81
Syringin	3.70	15.88 ± 1.55
Protocatechuic acid	5.03	259.50 ± 9.58
*α*-Resorcylic acid	5.76	4.34 ± 0.09
Rutin	6.22	131.25 ± 5.85
Neochlorogenic acid	6.74	21.58 ± 1.12
Chlorogenic acid	7.06	44.90 ± 1.98
*p*-Hydroxybenzoic acid	7.76	31.25 ± 0.72
Caffeic acid	8.65	ND
Syringic acid	9.99	3.98 ± 0.10
Vanillic acid	11.46	19.87 ± 0.72
Gentisic acid	12.01	2.52 ± 0.10
*p*-Coumaric acid	12.33	5.58 ± 0.15
(+) Catechin	12.89	152.23 ± 6.56
Ferulic acid	13.15	2.88 ± 0.09
Salicylic acid	13.45	2.98 ± 0.12
Isoquercitrin	15.05	65.80 ± 1.25
Myricetin	17.54	35.62 ± 0.69
Quercitrin	18.67	88.56 ± 2.12
Quercetin	24.78	52.55 ± 1.15

The measurements were performed in triplicates. Mean ± S.D. ND- not detected, below the limit of detection.

**Table 3:** Antioxidant activity of the *Omija* extract.

Parameter	Value

DPPH assay	5.93 mg TEAC/g d.w.
MRAP assay	140.52 mg TEAC/g d.w.

The measurements were performed in triplicates. Mean ± S.D.

**Table 4:** Antibacterial activity of the *Omija* extract against bacterial species tested by the disc diffusion assay.

Bacterial strains	Inhibition zones (mm)	
	Gentamicin	*Omija* extract

*Klebsiella pneumoniae*	4.33 ± 0.58	7.33 ± 2.08
*Pseudomonas aeruginosa*	4.00 ± 1.00	8.00 ± 1.00
*Salmonella enterica* subsp. *enterica*	5.00 ± 1.00	7.00 ± 2.65
*Shigella sonei*	5.67 ± 1.53	7.00 ± 1.00
*Yersinia enterocolitica*	5.33 ± 1.53	7.33 ± 2.08
*Bacillus cereus*	6.67 ± 1.53	9.00 ± 1.00
*Enterococcus faecalis*	6.00 ± 1.00	10.00 ± 2.00
*Listeria monocytogenes*	6.00 ± 1.73	8.00 ± 2.65
*Stapylococcus aureus* subsp. *aureus*	5.67 ± 1.53	8.00 ± 1.00
*Streptococcus pneumoniae*	6.67 ± 1.53	11.00 ± 1.00

The measurements were performed in triplicates. Mean ± S.D.

**Table 5:** Minimal Inhibition Concentration (MIC) of the *Omija* extract.

Bacterial strains	MIC 50 (*µ*g/mL)	MIC 90 (*µ*g/mL)

*Klebsiella pneumoniae*	128.1	136.4
*Pseudomonas aeruginosa*	130.6	143.8
*Salmonella enterica* subsp. *enterica*	170.7	192.9
*Shigella sonei*	128.1	136.4
*Yersinia enterocolitica*	170.7	192.9
*Bacillus cereus*	85.3	99.6
*Enterococcus faecalis*	64.2	72.2
*Listeria monocytogenes*	64.2	72.2
*Stapylococcus aureus* subsp. *aureus*	85.3	99.6
*Streptococcus pneumoniae*	85.3	99.6

The measurements were performed in eight replicates. Mean ± S.D.

**Table 6:** Bovine spermatozoa motility (%) in the absence (Ctrl) or presence of the *Omija* extract during different time periods.

Groups	0 h	2 h	24 h

Ctrl	86.25 ± 3.55	79.98 ± 2.04	20.44 ± 3.00
1 *µ*g/mL	85.90 ± 3.11	82.33 ± 2.05	50.00 ± 1.72^*∗∗∗*^
5 *µ*g/mL	84.00 ± 2.89	82.67 ± 2.35	51.22 ± 2.42^*∗∗∗*^
10 *µ*g/mL	91.44 ± 2.98	87.23 ± 2.22	55.54 ± 2.87^*∗∗∗*^
25 *µ*g/mL	90.09 ± 2.99	88.98 ± 1.17	50.95 ± 2.05^*∗∗∗*^
50 *µ*g/mL	88.33 ± 3.45	83.88 ± 2.07	45.03 ± 2.67^*∗∗∗*^
75 *µ*g/mL	82.33 ± 2.18	81.00 ± 2.90	18.00 ± 1.05

Mean ± S.D. (*n* = 30). ^*∗∗∗*^-*p* < 0.001.

**Table 7:** Reactive oxygen species (ROS) production by bovine spermatozoa (RLU/sec/10^6^ sperm) in the absence (Ctrl) or presence of the *Omija* extract during different time periods.

Groups	0 h	2 h	24 h

Ctrl	2.22 ± 0.92	4.66 ± 0.99	15.56 ± 1.99
1 *µ*g/mL	2.09 ± 0.67	4.00 ± 0.55	13.09 ± 1.44^*∗∗*^
5 *µ*g/mL	2.00 ± 0.48	3.60 ± 0.72^*∗*^	12.01 ± 1.76^*∗∗∗*^
10 *µ*g/mL	1.89 ± 0.62	3.55 ± 0.55^*∗*^	11.92 ± 1.34^*∗∗∗*^
25 *µ*g/mL	2.00 ± 0.90	3.08 ± 0.88^*∗∗*^	10.75 ± 1.49^*∗∗∗*^
50 *µ*g/mL	2.05 ± 0.69	3.77 ± 0.71^*∗*^	12.88 ± 1.77^*∗∗*^
75 *µ*g/mL	2.75 ± 0.83	5.10 ± 0.98	16.89 ± 1.98

Mean ± S.D. (*n* = 30). ^*∗∗∗*^-*p* < 0.001; ^*∗∗*^-*p* < 0.01; ^*∗*^-*p* < 0.05.

**Table 8:** Intracellular total antioxidant capacity of bovine spermatozoa (eq. *μ*mol Trolox/g prot) in the absence (Ctrl) or presence of the *Omija* extract during different time periods.

Groups	0 h	2 h	24 h

Ctrl	4.07 ± 0.48	3.90 ± 0.23	2.12 ± 0.19
1 *µ*g/mL	4.10 ± 0.52	4.44 ± 0.65^*∗∗*^	3.19 ± 0.19^*∗∗*^
5 *µ*g/mL	4.12 ± 0.27	5.01 ± 7.25^*∗∗*^	3.55 ± 0.55^*∗∗*^
10 *µ*g/mL	4.26 ± 0.39	5.48 ± 0.46^*∗∗*^	4.82 ± 0.32^*∗∗*^
25 *µ*g/mL	4.20 ± 0.34	5.55 ± 0.41^*∗∗*^	4.88 ± 0.57^*∗∗*^
50 *µ*g/mL	4.16 ± 0.55	5.57 ± 0.35^*∗∗*^	3.39 ± 0.23^*∗∗*^
75 *µ*g/mL	4.10 ± 0.50	5.77 ± 0.42^*∗∗*^	3.22 ± 0.19^*∗∗*^

Mean ± S.D. (*n* = 30). ^*∗∗*^-*p* < 0.01.

**Table 9:** Protein oxidation expressed as the concentration of protein carbonyls assessed in bovine spermatozoa (nmol PC/mg prot) in the absence (Ctrl) or presence of the *Omija* extract during different time periods.

Groups	0 h	2 h	24 h

Ctrl	1.22 ± 0.15	2.63 ± 0.20	27.71 ± 0.90
1 *µ*g/mL	1.17 ± 0.12	1.83 ± 0.20^*∗*^	16.81 ± 0.67^*∗∗∗*^
5 *µ*g/mL	1.05 ± 0.12	1.66 ± 0.13^*∗∗∗*^	13.51 ± 0.65^*∗∗∗*^
10 *µ*g/mL	1.10 ± 0.15	1.71 ± 0.17^*∗∗∗*^	13.05 ± 0.60^*∗∗∗*^
25 *µ*g/mL	1.05 ± 0.13	1.56 ± 0.15^*∗∗∗*^	11.31 ± 0.55^*∗∗∗*^
50 *µ*g/mL	1.15 ± 0.14	1.76 ± 0.19^*∗*^	12.55 ± 0.59^*∗∗∗*^
75 *µ*g/mL	1.01 ± 0.10	1.72 ± 0.23^*∗*^	16.55 ± 0.76^*∗∗∗*^

Mean ± S.D. (*n* = 30). ^*∗∗∗*^-*p* < 0.001; ^*∗*^-*p* < 0.05.

**Table 10:** Lipid peroxidation expressed as the malondialdehyde content (*μ*mol MDA/g prot) assessed in bovine spermatozoa in the absence (Ctrl) or presence of the *Omija* extract during different time periods.

Groups	0 h	2 h	24 h

Ctrl	0.33 ± 0.04	0.93 ± 0.05	7.18 ± 0.24
1 *µ*g/mL	0.30 ± 0.02	0.48 ± 0.03^*∗∗∗*^	4.26 ± 0.23^*∗∗∗*^
5 *µ*g/mL	0.28 ± 0.03	0.43 ± 0.02^*∗∗∗*^	3.48 ± 0.20^*∗∗∗*^
10 *µ*g/mL	0.25 ± 0.04	0.37 ± 0.02^*∗∗∗*^	2.56 ± 0.19^*∗∗∗*^
25 *µ*g/mL	0.29 ± 0.03	0.42 ± 0.03^*∗∗∗*^	3.02 ± 0.21^*∗∗∗*^
50 *µ*g/mL	0.30 ± 0.02	0.53 ± 0.04^*∗∗∗*^	5.40 ± 0.35^*∗∗∗*^
75 *µ*g/mL	0.32 ± 0.02	0.59 ± 0.04^*∗∗∗*^	6.02 ± 0.30^*∗∗∗*^

Mean ± S.D. (*n* = 30). ^*∗∗∗*^-*p* < 0.001.

## Data Availability

The data used to support the findings of this study are available from the corresponding author upon request.
